# Association of Serum Vitamin D With Macular Microvascular Structure in Type 2 Diabetic Mellitus Without Diabetic Retinopathy: A Cross‐Sectional Study

**DOI:** 10.1155/jdr/5539240

**Published:** 2026-01-18

**Authors:** Hui Yang, Xinyan Ma, Youjin Pan, Suilian Zheng, Haihua Zheng, Zheren Xia

**Affiliations:** ^1^ Department of Ophthalmology, The Second Affiliated Hospital of Wenzhou Medical University, Zhejiang, China, wmu.edu.cn; ^2^ Department of Ophthalmology, The Wenzhou People’s Hospital, Zhejiang, China; ^3^ Department of Endocrinology, The Second Affiliated Hospital of Wenzhou Medical University, Zhejiang, China, wmu.edu.cn

**Keywords:** optical coherence tomography angiography, perfusion density, retinal microvasculature, Type 2 diabetes, vitamin D

## Abstract

**Purpose:**

This study investigated the association of different serum vitamin D levels on macular microvascular structure in Type 2 diabetes mellitus (T2DM) patients without diabetic retinopathy (DR), utilizing optical coherence tomography angiography (OCTA).

**Methods:**

A cross‐sectional observational study was designed that includes 83 patients (83 eyes) with T2DM but without DR. Three groups were defined based on vitamin D levels, including vitamin D deficiency (serum vitamin D < 20 ng/mL), vitamin D insufficiency (20 ng/mL ≤ serum vitamin D < 30 ng/mL), and normal vitamin D (≥ 30 ng/mL) groups. All patients underwent OCTA with a 6 × 6‐mm scan centered on the fovea. Assessment was conducted on the perfusion density (PD) of the retinal full layer (FL) and the superficial capillary plexus (SCP). In addition, evaluation of the foveal avascular zone (FAZ) included its area, perimeter, and acircularity index (AI).

**Results:**

The PD of the SCP was significantly lower in the vitamin D deficiency group, especially in the temporal regions (42.04 ± 3.8 vs. 44.94 ± 4.5 vs. 47.24 ± 3.9, *p* < 0.001). The PD of the retinal FL was also significantly lower in the vitamin D deficiency group perifoveal area (32.94 ± 4.5 vs. 35.75 ± 4.2 vs. 35.57 ± 4.4, *p* < 0.05).

**Conclusions:**

Changes in PD were found to be particularly sensitive to changes in the temporal‐perifoveal region in individuals with vitamin D deficiency. Thus, vitamin D deficiency may be associated with DR.

## 1. Introduction

Diabetes mellitus (DM) has become a major issue in the realm of global health with high rates of morbidity and mortality [1]. Beyond the detrimental effects of diabetes on daily life, it also has long‐term consequences, such as diabetic retinopathy (DR), which results in a diminished quality of life. DR is among the most prevalent diabetes complications and affects a sizable proportion of individuals with diabetes. Severe DR can result in vision impairment and blindness [2].

Early screening for DR has been shown to reduce both the prevalence and disease severity in patients with diabetes, highlighting the importance of early assessment, prediction, and intervention before DR develops [3]. Histopathological studies have demonstrated that changes in retinal vasculature occur before the clinical signs of DR become apparent [4]. Therefore, assessing changes in retinal blood flow could serve as a novel clinical method for predicting DR progression and prognosis, potentially preventing vision loss in affected patients.

Optical coherence tomography angiography (OCTA) is a newly emerged, noninvasive imaging modality that has become pivotal for both qualitative and quantitative assessment of the retinal vascular system. It is widely used to assess DR severity. Many studies have analyzed OCTA imaging features—such as the perfusion density (PD), foveal avascular zone (FAZ), fractal dimension, and nonperfusion area—most of which are closely associated with early DR. These features have shown significant diagnostic accuracy and predictive value for DR progression [5]. Retinal vessels are among the few blood vessels in the body that can be directly observed. Quantitative studies of the retinal vasculature using OCTA to predict DR severity and identify high‐risk patients could represent a new strategy in clinical practice.

Vitamin D is of vital significance in vascular protection and regeneration. An expanding body of research suggests that vitamin D deficiency (VDD) is correlated with diabetes and DR. [6] Research has revealed an inverse relationship between the occurrence and severity of DR and vitamin D levels [7]. Luo et al. [8] confirmed that individuals with Type 2 diabetes mellitus (T2DM) and VDD, indicated by serum 25(OH)D levels below 20 ng/mL, face a substantially higher risk of developing and progressing DR. However, whether the serum vitamin D level is related to retinal blood flow in patients with diabetes remains an unaddressed knowledge gap.

This study is aimed at evaluating the relationship between serum vitamin D levels and macular microvascular parameters in T2DM patients without clinical DR using OCTA.

## 2. Methods

### 2.1. Study Design and Oversight

This study included 83 T2DM patients without DR, who were recruited from the Endocrinology Department of the Second Affiliated Hospital of Wenzhou Medical University. According to standard criteria [9], patients were categorized into three groups according to their vitamin D levels: Group A (VDD; serum vitamin D < 20 ng/mL, *n* = 26), Group B (vitamin D insufficiency; 20 ng/mL ≤ serum vitamin D < 30 ng/mL, *n* = 30), and Group C (normal vitamin D; serum vitamin D ≥ 30 ng/mL, *n* = 27). Stata 13.1 was used to calculate the sample size to ensure sufficient power. All patients met the diagnostic criteria for T2DM (HbA1c > 6.5% and fasting plasma glucose > 7 mmol/L) and had no evidence of DR when examined by ultrawide–field color fundus photography using an Optos Daytona ultrawide–field fundus camera [10, 11]. The Ethics Committee of Wenzhou Medical University approved the study. All participants gave written informed consent, and the study followed the Declaration of Helsinki principles.

### 2.2. Patient Recruitment

The inclusion criteria were patients with Type 2 diabetes without DR. Participants with the following conditions were excluded: (1) other retinal diseases; (2) history of intraocular surgery or ocular trauma; (3) chronic or recurrent uveitis or chronic open‐angle or angle‐closure glaucoma; (4) factors that may affect image quality, such as media opacities, nystagmus, or poor fixation or cooperation; (5) uncontrolled systemic hypertension; (6) ketoacidosis; (7) chronic kidney disease (24‐h proteinuria > 0.2 g/mL), active infection (hypersensitive C‐reactive protein > 10 mg/L), infectious liver disease, primary or secondary hyperparathyroidism (< 70 pg/mL), or a serum creatinine level of > 176.8 mg/dL; (8) current use of vitamin D or calcium supplements, as well as medications that affect vitamin D metabolism (e.g., rifampin or phenytoin); and (9) history of fractures or orthopedic surgery within the past year or history of pituitary adenoma surgery.

### 2.3. Ophthalmic Examination

Clinical examination and laboratory analyses confirmed a diagnosis of diabetes. Baseline ophthalmic checks, including slit lamp, fundus examinations, axial length, intraocular pressure, and refraction measurements, were performed by the same ophthalmologist, and the ophthalmologist in charge of the eye examination was unaware of the patient′s serum vitamin D levels, glycated hemoglobin, and other relevant information.

Data collected included age, body mass index (BMI), sex, duration of diabetes, HbA1c levels, and serum 25(OH)D levels, which were evaluated using electrochemiluminescence.

OCTA was performed using an SS‐OCTA system (VG100N, Intalight, China), which is capable of performing A‐scans at a rate of 100,000 scans per second, with a 6 × 6‐mm scan centered on the fovea. Images with a signal strength index higher than eight were included in the analysis. The macular region was automatically partitioned into the foveal region (0–1 mm), parafoveal region (1–3 mm), perifoveal region (3–6 mm), and four quadrants (temporal, nasal, superior, and inferior) (Figure 1). All of the patients were pharmacologically dilated using tropicamide and then scanned using the same OCTA machine with the same mode, and this process was repeated three times. The OCTA images were reviewed by an experienced retinal specialist, and segment errors (such as the lines not corresponding to the proper retinal layers) or images of low quality, if any, were excluded. Based on the optical design of the device, the magnification correction formula was optimized from the Bennett′s formula [12]. The magnification for fundus measurement is given by: Magnification = 0.0492 × AL − 0.1818, where a default axial length (AL) of 24 mm corresponds to a fundus magnification of 1. The resultant data were automatically calculated by the integrated software subsequent to the correction for AL. The statistical values were calculated as the average of the three repetitions.

Figure 1Representative optical coherence tomography angiography (OCTA) images of perfusion density (PD) of the superficial capillary plexus (SCP) and inner retinal layer (IRL) foveal avascular zone (FAZ). The macular region was subdivided into the foveal area (0–1 mm), parafoveal area (1–3 mm), and perifoveal area (3–6 mm). Nasal (N), superior (S), temporal (T), and inferior (I) regions are shown in the perifoveal circular region (a). Colored PD map of the SCP (b). Perfusion area (PA) map of the SCP (c). FAZ map of the IRL (d).

**Figure:**
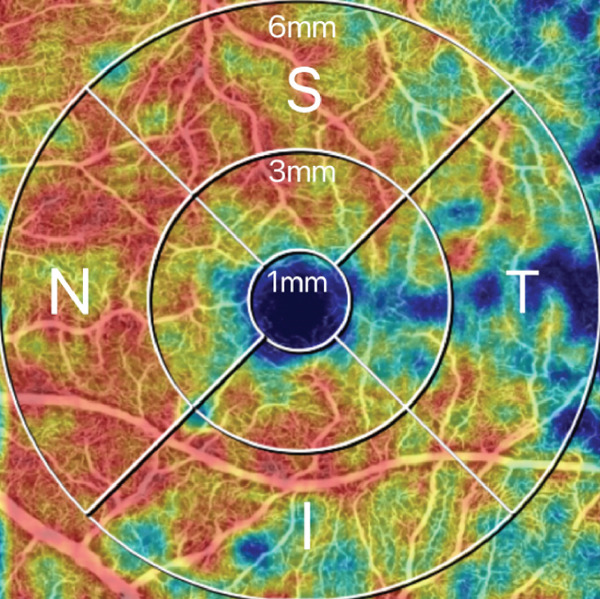
(a)

**Figure:**
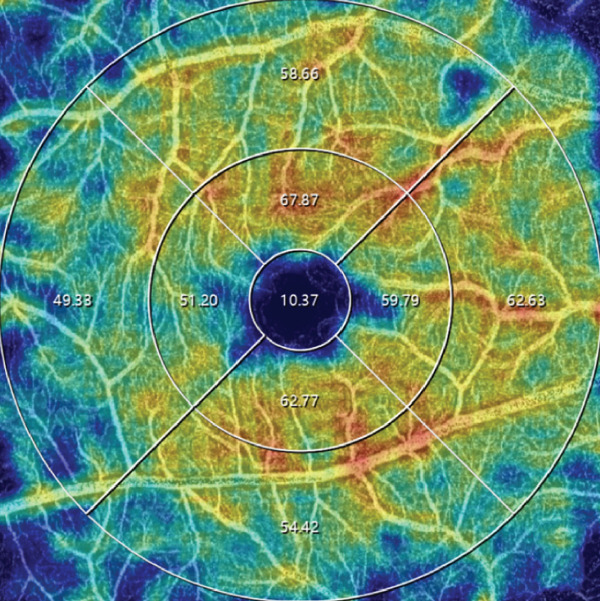
(b)

**Figure:**
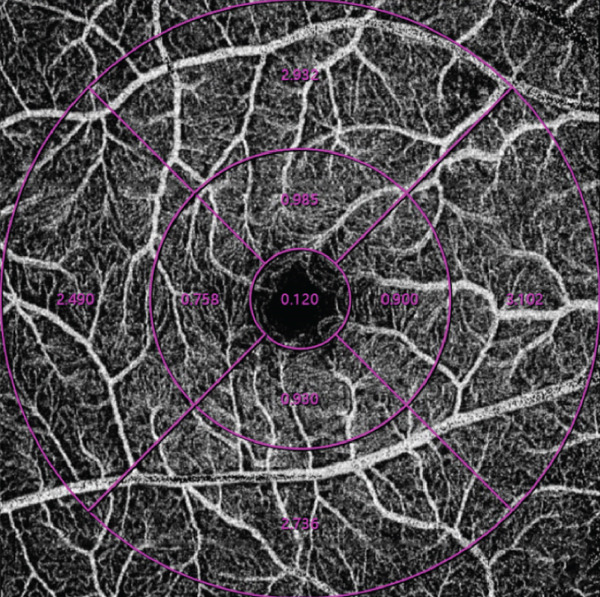
(c)

**Figure:**
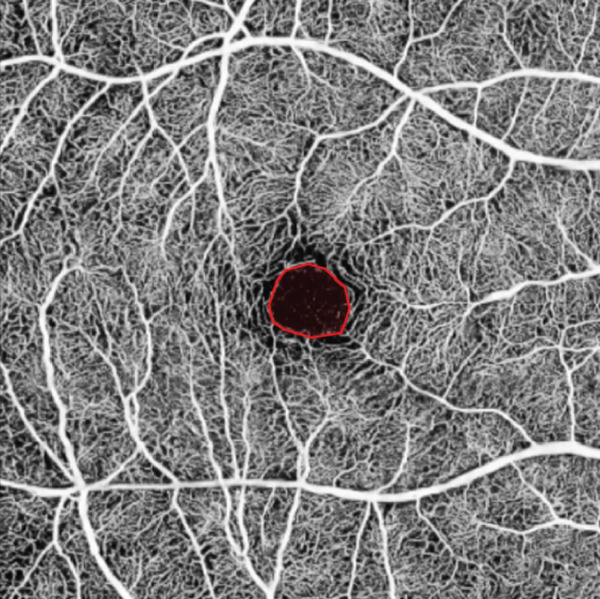
(d)

For all the patients, the PD of the retinal full layer (FL) and the superficial capillary plexus (SCP) was measured along the four quadrants. FAZ‐related parameters, such as perimeter, area, and acircularity index (AI) in the inner retinal layer (IRL) were assessed. All OCTA examinations were conducted by the same ophthalmologist to maintain consistency. Representative OCTA images of the PD of the SCP and IRL FAZ are shown in Figure 1.

### 2.4. Statistical Analysis

IBM SPSS (Version 27.0) was employed to conduct the statistical analyses. The Kolmogorov–Smirnov test was used to assess data normality. One‐way analysis of variance was used to analyze normally distributed data, whereas the nonparametric Kruskal–Wallis test was used to analyze nonnormally distributed data. The least significant difference method was used to analyze pairwise comparison, and statistical significance was indicated by *p* < 0.05. The analyses compared vitamin D levels, age, BMI, diabetes duration, HbA1c level, axial length, and OCTA imaging features across the groups. The chi‐square test assessed the significance of differences in sex distribution among the groups. Pearson′s correlation analysis was conducted for the vitamin D and OCTA parameters.

## 3. Results

The medical and demographic features are summarized in Table 1. When comparing the groups, no statistically significant differences were found in age, BMI, diabetes duration, HbA1c levels, or axial length (*p* > 0.05). The mean serum 25(OH)D levels were 15.8 ± 3.4 ng/mL in Group A, 25.0 ± 2.8 ng/mL in Group B, and 36.8 ± 5.9 ng/mL in Group C. Statistically significant differences in serum 25(OH)D levels were characterized among the groups (*p* < 0.001).

**Table 1:** Clinical and demographic characteristics of all groups.

	**Group A** **n** = 26	**Group B** **n** = 30	**Group C** **n** = 27	**p**
Age, mean ± SD, *y*	48.0 ± 16.3	53.6 ± 12.6	56.9 ± 13.3	0.126
Male,%	51.9%	60.0%	65.4%	0.600
Course,^a^ *y*	5.2 ± 5.7	6.5 ± 6.0	7.2 ± 6.3	0.416
Hb1Ac^b^ level, %	9.5 ± 2.9	8.6 ± 2.8	8.4 ± 2.3	0.267
BMI	24.2 ± 3.5	24.4 ± 3.5	24.9 ± 3.2	0.750
Eye axis	24.06 ± 1.0	23.57 ± 1.1	23.63 ± 1.2	0.194
25(OH)D (ng/mL)	14.9 ± 3.8	25.0 ± 2.7	36.8 ± 6.2	< 0.001∗

*Note:* Chi‐square test was used for male% analysis and one‐way analysis of variance was used for the other data. Data are means ± SD.

Abbreviation: BMI, body mass index.

^a^Course: Duration of diabetes.

^b^HbA1c, glycated hemoglobin.

∗*p* < 0.05, considered statistically significant between the three groups.

At the SCP layer, patients with VDD had a lower PD than those with vitamin D insufficiency. The differences were significant in the temporal and superior quadrants. Furthermore, vitamin D–deficient patients have a significantly lower PD than vitamin D–sufficient patients in all regions. (Table 2).

**Table 2:** Comparison of PD of the SCP in different subregions.

**PD, %**	**Group A**	**Group B**	**Group C**	**p** **(AvsB)**	**p** **(AvsC)**	**p** **(BvsC)**
1–6 mm	42.04 ± 3.8	44.94 ± 4.5	47.24 ± 3.9	0.009∗	< 0.001∗	0.038∗
1–3 mm	45.76 ± 5.1	50.28 ± 6.1	51.29 ± 4.6	0.002∗	< 0.001∗	0.483
3–6 mm	40.93 ± 4.4	43.33 ± 4.8	46.04 ± 4.2	0.048∗	< 0.001∗	0.027∗
1–6 mm T	36.62 ± 5.7	41.23 ± 5.2	41.60 ± 4.3	0.001∗	< 0.001∗	0.784
1–3 mm T	40.72 ± 6.0	46.26 ± 5.7	47.26 ± 5.4	< 0.001∗	< 0.001∗	0.515
3–6 mm T	35.39 ± 6.3	39.72 ± 6.0	39.91 ± 4.6	0.006∗	0.005∗	0.901
1–6 mm S	43.07 ± 4.9	46.88 ± 5.9	49.97 ± 5.1	0.009∗	< 0.001∗	0.034
1–3 mm S	47.28 ± 5.1	52.96 ± 6.5	53.99 ± 5.2	< 0.001∗	< 0.001∗	0.498
3–6 mm S	41.82 ± 5.9	45.08 ± 6.7	48.78 ± 5.6	0.049∗	< 0.001∗	0.027∗
1–6 mm N	46.88 ± 4.9	49.10 ± 6.5	51.91 ± 4.7	0.129	0.001∗	0.06
1–3 mm N	45.32 ± 6.4	48.83 ± 7.8	49.82 ± 5.9	0.056	0.018∗	0.586
3–6 mm N	47.34 ± 5.7	49.18 ± 6.6	52.53 ± 5.1	0.241	0.002∗	0.037∗
1–6 mm I	41.37 ± 5.3	42.51 ± 5.3	45.43 ± 4.6	0.402	0.005∗	0.036∗
1–3 mm I	49.71 ± 5.9	53.07 ± 7.1	54.08 ± 5.2	0.044∗	0.012∗	0.542
3–6 mm I	39.05 ± 5.6	39.28 ± 5.8	42.77 ± 5.2	0.878	0.017∗	0.022∗

*Note:* The grouped *t*‐test was used for pairwise comparisons among the three groups. Data are means ± SD.

Abbreviations: I, inferior region; N, nasal region; PD, perfusion density; S, superior region; SCP, superficial capillary plexus; T, temporal region.

∗*p* < 0.05, considered statistically significant between the groups.

Further analysis of the retinal FL 3–6 mm region revealed that Group A had a significantly lower PD than both Groups B and C, especially in the superior region (*p* < 0.05). In contrast, differences in PD between Groups B and C were not statistically significant (*p* > 0.05) (Table 3).

**Table 3:** Comparison of PD of the retinal FL in different subregions.

**PD, %**	**Group A**	**Group B**	**Group C**	**p** **(AvsB)**	**p** **(AvsC)**	**p** **(BvsC)**
1–6 mm	27.44 ± 3.9	29.74 ± 3.6	29.46 ± 3.8	0.023∗	0.054	0.781
1–3 mm	9.16 ± 2.5	9.75 ± 2.7	9.15 ± 2.7	0.401	0.995	0.402
3–6 mm	32.94 ± 4.5	35.75 ± 4.2	35.57 ± 4.4	0.018∗	0.032∗	0.879
1–6 mm T	14.48 ± 7.2	16.52 ± 6.7	15.27 ± 5.6	0.244	0.662	0.478
1–3 mm T	5.95 ± 4.5	5.52 ± 3.6	5.23 ± 4.8	0.706	0.545	0.805
3–6 mm T	17.05 ± 8.1	19.81 ± 7.8	18.28 ± 6.0	0.163	0.546	0.442
1–6 mm S	30.31 ± 4.7	33.12 ± 5.1	33.20 ± 5.0	0.035∗	0.036∗	0.954
1–3 mm S	11.06 ± 3.0	12.56 ± 3.6	12.26 ± 3.5	0.096	0.196	0.743
3–6 mm S	35.02 ± 7.3	39.22 ± 6.4	37.91 ± 6.8	0.019∗	0.018∗	0.916
1–6 mm N	33.54 ± 6.3	36.07 ± 6.3	35.10 ± 6.3	0.133	0.237	0.799
1–3 mm N	6.80 ± 4.3	7.23 ± 2.9	6.20 ± 2.5	0.630	0.511	0.251
3–6 mm N	41.47 ± 7.2	44.73 ± 7.8	43.54 ± 7.6	0.106	0.174	0.834
1–6 mm I	31.45 ± 4.3	33.33 ± 4.2	33.81 ± 4.2	0.098	0.046∗	0.674
1–3 mm I	12.83 ± 3.6	13.69 ± 3.9	12.93 ± 3.3	0.375	0.924	0.435
3–6 mm I	37.18 ± 5.1	39.36 ± 5.0	40.35 ± 5.1	0.109	0.025∗	0.466

*Note:* The grouped *t*‐test was used for pairwise comparisons among the three groups. Data are means ± SD.

Abbreviations: FL, full layer; I, inferior region; N, nasal region; PD, perfusion density; S, superior region; T, temporal region.

∗ p <0.05, considered statistically significant between the groups.

The differences in PD of the FL 1–3 mm region, FAZ area, perimeter, and AI were not statistically significant among the three groups (*p* > 0.05) (Tables 3 and 4).

**Table 4:** Comparison of FAZ area, perimeter, and AI of IRL in the three groups.

**FAZ**	**Group A**	**Group B**	**Group C**	**p**
Area, mm^2^	0.32 ± 0.1	0.34 ± 0.1	0.39 ± 0.1	0.157
Perimeter, mm	2.32 ± 0.5	2.37 ± 0.6	2.48 ± 0.4	0.555
AI	0.780 ± 0.1	0.781 ± 0.1	0.784 ± 0.1	0.967

*Note:* One‐way analysis of variance was used for the data in this table. Data are means ± SD; *p* < 0.05 was considered statistically significant between the three groups.

Abbreviations: AI, acircularity index; FAZ, foveal avascular zone.

The Pearson correlation analysis revealed that except for the inferior and nasal area of the 1–3 mm region, the SCP showed a weak positive correlation with vitamin D in all other regions. The PD in the inferior area of 3–6 mm of the FL had a weak correlation with vitamin D. The area of the FAZ was weakly correlated with vitamin D (Table 5).

**Table 5:** Correlation analysis of serum vitamin D levels and OCTA parameters.

	**SCP parameters**		**FL parameters**	
	**r**	**p**	**r**	**p**
1–6 mm	0.381	< 0.001∗	0.186	0.092
1–3 mm	0.252	0.021∗	0.005	0.962
3–6 mm	0.371	0.001∗	0.206	0.061
1–6 mm T	0.281	0.010∗	0.073	0.511
1–3 mm T	0.289	0.008∗	−0.018	0.875
3–6 mm T	0.243	0.027∗	0.088	0.431
1–6 mm S	0.400	<0.001∗	0.146	0.187
1–3 mm S	0.290	0.008∗	0.084	0.449
3–6 mm S	0.382	<0.001∗	0.163	0.140
1–6 mm N	0.284	0.009∗	0.109	0.326
1–3 mm N	0.159	0.151	−0.029	0.794
3–6 mm N	0.290	0.008∗	0.119	0.284
1–6 mm I	0.273	0.013∗	0.218	0.047∗
1–3 mm I	0.170	0.125	−0.016	0.887
3–6 mm I	0.247	0.024∗	0.250	0.023∗
FAZ area			0.244	0.026∗
FAZ perimeter			0.195	0.078
FAZ AI			−0.028	0.799

*Note:* Person’s correlation analysis was used for the data.

Abbreviations: AI, acircularity index; FAZ, foveal avascular zone; FL, full layer; I, inferior region; N, nasal region; S, superior region; SCP, superficial capillary plexus; T, temporal region.

∗*p* < 0.05, considered statistically significant.

## 4. Discussion

DR is a significant diabetes‐related complication that can greatly impact vision, particularly when DR progresses to severe nonproliferative diabetic retinopathy or PDR, which greatly increases the risk of vision loss [2]. Current theories suggest that T2DM induces various mechanisms, such as oxidative stress, immune responses, and chronic inflammation, leading to a reduction in retinal PD. This results in retinal ischemia and hypoxia, which in turn triggers the upregulation of vascular endothelial growth factor and other angiogenic factors, accelerating the progression of DR to PDR [13]. Research has indicated that VDD not only contributes to the onset of DR but also serves as a potential factor in its progression [14]. Vitamin D exerts protective effects against DR development and progression by acting on vitamin D receptors (VDRs) expressed in retinal endothelial cells (ECs), pericytes (PCs), and vascular smooth muscle cells through various mechanisms, such as reducing oxidative stress, apoptosis, and inflammation [15, 16]. Research has shown that angiopoietin‐2 (Ang2) in retinal PCs of diabetic mice triggers PC apoptosis through the p53 signaling pathway [17]. In addition, increased vitamin D levels have been reported to reduce Ang2 expression in the kidneys and lungs [18]. Therefore, we hypothesized that vitamin D may also reduce Ang2 expression in retinal VDR, thereby protecting against PC apoptosis. However, further research is required to validate this hypothesis.

Prior studies on VDD and retinal vascular density have yielded inconsistent findings. A prospective study has reported that in patients with VDD, significant decreases in vascular density were observed in the SCP, deep capillary plexus (DCP), and FAZ [19]. However, an observational case–control study found that a deficiency of serum vitamin D can lead to an increase in vascular density, especially in DCPs [20]. These discrepant results may be attributed to variations in study populations, research designs, and other methodological factors. Nevertheless, all of these studies were based on patients without diabetes and no research on the association between vitamin D and retinal microvessels in patients with T2DM has been undertaken to date.

In this study, we used a 6 × 6‐mm OCTA scan to quantitatively analyze the impact of vitamin D levels on macular microvascular structure, including the retinal FL and SCP PD, as well as FAZ‐related parameters, such as the area, perimeter, and AI of T2DM patients without DR.

The findings of our research showed that the PD of the SCP in all regions was significantly lower in Group A compared with Groups B and C, suggesting that VDD is associated with a notable decrease in macular vessel density. This could stem from vitamin D′s diminished protective impact on the retinal microvasculature. Retinal microvessels are primarily composed of ECs and PCs, both of which are involved in vascular contraction [13]. Studies have shown that VDR expression is significantly higher in PCs compared with ECs [21], and that hyperglycemia‐induced PC apoptosis can lead to the disruption of capillary homeostasis, further affecting EC survival and leading to capillary closure, thereby reducing retinal microvascular density [22].

Our research revealed a statistically significant difference in PD between Groups B and C, especially in the perifoveal area (3–6 mm), suggesting that VDD is also associated with a lower density of retinal vascular perfusion. Correlation analysis results showed a positive correlation between vitamin D and perifoveal area PD. This finding corresponds to the results of Castillo‐Otí et al. [23], who reported that lower serum 25(OH)D levels were linked to a higher risk of DR onset and progression in T2DM patients.

Our study found that the PD of the temporal region in the three subfields was significantly lower in Group A than in Groups B and C, with the reduction in PD in the SCP being particularly pronounced in this region in patients with VDD. This suggests that ischemia is more likely to occur in the temporal region in patients with VDD. In addition, in this study, PD was significantly lower in the perifoveal area (3–6 mm) in Group A than in the other two groups, suggesting that microvascular changes in patients with VDD may initially occur in this area. This suggests that changes in retinal blood flow may be most sensitive in the perifoveal‐temporal region, a finding that aligns with that of Alam et al. [5], who identified the temporal‐perifoveal region as the most sensitive area for the early detection of DR.

Structural changes of FAZ, primarily characterized by an increase in area and perimeter, are caused by the loss of capillaries in the fovea. In this study, although FAZ structural changes and a slight downward trend in AI were observed in the three groups with decreasing vitamin D levels, resulting in a tendency toward an irregular FAZ shape, no significant differences in these parameters were identified among the groups. FAZ size varies among individuals and is influenced by multiple factors such as age, sex, central macular thickness, and axial length. In addition, variations in measurement data from different devices affect FAZ size estimates as well as errors due to different boundary segmentation methods. However, some studies have suggested that changes in FAZ size can serve as an indicator of retinal microvascular ischemia. [24, 25] Therefore, comparisons of FAZ size should take these factors into account, and the FAZ may not be suitable as an early screening indicator of DR.

As for the AI, a previous study suggested that it may better represent microvascular changes than FAZ size [25], making it potentially useful for early screening. AI reflects irregularities in the FAZ shape and serves as an indicator of capillary loss and macular ischemia. However, our study revealed no statistically significant differences in AI among the three groups. This suggests that AI may not be a sensitive indicator of microvascular changes caused by VDD. However, further research with a more extensive sample size is needed to confirm this conclusion.

In our study, we utilized SS‐OCTA, which offers the advantages of a faster scanning speed and a larger scanning area. With its longer wavelength and higher sensitivity, SS‐OCTA enhances penetration through the RPE, allowing for better detection of deeper blood flow signals [26]. Many previous DR studies have focused on 3 × 3‐mm OCTA images. In contrast to OCTA, SS‐OCTA enables scanning of a larger area, such as beyond the posterior pole, in a single acquisition. According to de Carlo et al., the 6 × 6‐mm scan mode, with its larger field of view, is more effective in detecting retinal blood flow changes associated with DR than the 3 × 3‐mm scan mode [27]. Wide‐field imaging may be beneficial in assessing the retinal vascular system in patients with DR. However, this mode requires higher patient compliance and has a lower resolution than the other two modes [28].

Previous research has shown that the DCP responds earlier and more prominently to hyperglycemia [29]. However, as noise and artifacts from shallow blood flow or refractive media may affect the detection of deep blood flow [7], deeper retinal vessels were not investigated in the current study. Instead, the analysis was limited to specific retinal regions, which may not fully represent changes in retinal blood flow. Future research with larger sample sizes and OCTA imaging of deeper retinal layers is needed to achieve a more thorough understanding of the relationship between vitamin D and retinal blood flow and perfusion in diabetic patients.

The observed variability in serum 25‐hydroxyvitamin D levels among our cohort of diabetic patients is likely multifactorial. Several factors, many of which are highly prevalent in this population, could contribute to this heterogeneity: differences in sun exposure, adiposity, dietary and supplemental intake, diabetic nephropathy, and genetic factors. We acknowledge that these very factors, particularly adiposity, dietary habits, and the presence/severity of nephropathy, could also be independent confounding variables that directly influence retinal microvasculature and OCTA metrics. Although this study controlled for some lurking variables, such as age, BMI, and HbA1c, it remains a possibility that the association we observed is not solely driven by vitamin D itself but may be mediated or confounded by these other pathophysiological elements of diabetes. Future studies with longitudinal designs and more comprehensive covariate adjustments are needed to disentangle the specific role of vitamin D from other closely related metabolic and lifestyle factors.

This study has several limitations that should be considered when interpreting the results. First, the cross‐sectional nature of the design precludes the determination of causal relationships between the variables examined. Second, the findings are based on a relatively small sample size, which may have limited the statistical power to detect significant associations. Third, the study lacked a direct evaluation of the DCP, which could have provided a more comprehensive analysis. Furthermore, potential confounding factors, such as dietary intake, sun exposure, and physical activity, were not fully accounted for and may have influenced the outcomes. A further limitation is the reliance on a single measurement of serum vitamin D levels. Although this provides a snapshot of vitamin D status, it may not fully represent long‐term or historical exposure, which could be more pertinent to the development of chronic retinal microvascular changes. Finally, the specific characteristics of the study population may limit the generalizability of our findings to other broader or different demographic groups.

This study demonstrated an association between lower serum vitamin D levels and reduced macular PD, particularly in the perifoveal‐temporal region, in patients with Type 2 diabetes without clinical signs of DR. Future longitudinal studies with repeated measures of vitamin D over time are needed to more accurately determine the relationship between sustained vitamin D status and the progression of diabetic retinal microvasculopathy.

## Ethics Statement

All measurements performed in this study involving human participants were in accordance with the ethical standards of the Institutional and National Research Committee and the Declaration of Helsinki. The study protocol was approved by the Ethics Committee of the Second Affiliated Hospital of the Wenzhou Medical University (ID: 2021133). All participants were informed of the issues related to this study and provided written informed consent.

## Consent

The participants understood and consented to submission to the journal. The authors obtained all appropriate participant consent forms.

## Disclosure

All authors have read and approved the final manuscript.

## Conflicts of Interest

The authors declare no conflicts of interest.

## Author Contributions

H.Y. and X.M. contributed equally to this study. Z.X., S.Z., and H.Z. contributed to the study design and protocol. Y.P. collected the data. H.Y. and X.M. measured and analyzed the data and performed ophthalmic examinations. H.Y., X.M., and Z.X. prepared and reviewed the manuscript.

## Funding

This study was supported by the Zhejiang Provincial Basic Public Welfare Research Project (No. LGC22H120001).

## Data Availability

The datasets created and/or analyzed during the current study are available from the corresponding author upon reasonable request.
